# Evaluation of antihypertensive potential of *Ficus carica* fruit

**DOI:** 10.1080/13880209.2017.1278611

**Published:** 2017-02-10

**Authors:** Alamgeer, Shifa Iman, Hira Asif, Muhammad Saleem

**Affiliations:** a Department of Pharmacology, Faculty of Pharmacy, University of Sargodha, Sargodha, Pakistan;; b Department of Pharmacy GC, University Faisalabad, Faisalabad, Pakistan

**Keywords:** Hypertension, flavonoids content, HPLC

## Abstract

**Context:** *Ficus carica* L. (Moraceae) fruit is said to possess cardiovascular activity and has been used empirically in traditional phytotherapies for the treatment of hypertension and various other cardiovascular diseases.

**Objective:** This study investigated the antihypertensive and cardioinhibitory activity of the aqueous-methanol extract of *F. carica* fruit in rats.

**Materials and methods:** Extract in 250, 500 and 1000 mg/kg doses (p.o.) were administered to normotensive Sprague Dawley rats and blood pressure was measured using non-invasive technique. Hypertension was induced in rats by oral administration of 10% glucose for 3 weeks. Hypotensive effect of extract (1000 mg/kg p.o) was studied in normotensive and glucose-treated hypertensive rats. Langendorff’s isolated heart technique was used to assess the effect of crude extract on force of contraction and heart rate. In addition, antioxidant potential, TPC, TFC were also assessed by DPPH free radical scavenging activity, Folin–Ciocalteu reagent and AlCl_3_ assay, respectively. Furthermore, phenolic compounds were analyzed using HPLC-DAD technique.

**Results and discussion:** The 1000 mg/kg dose decreased blood pressure significantly in normotensive and glucose-treated hypertensive rats. The isolated heart study showed that the extract produced negative inotropic and chronotropic effects but it failed to block the stimulatory effect of both adrenaline and CaCl_2_. HPLC studies on the *F. carica* extract indicated the presence of quercetin, gallic acid, caffeic acid, vanillic acid, syringic acid, coumaric acid and chromotropic acid.

**Conclusions:** This study demonstrated that aqueous methanol extract of *F. carica* fruit exerted hypotensive and antihypertensive effects in glucose-induced hypertensive rats.

## Introduction

All over the world cardiovascular diseases have remained one of the leading cause of death, hypertension being the most common and the main contributor to the pathogenesis of myocardial infarction, stroke and renal diseases (D’Agostino et al. 2008). About 600 million patients suffer from hypertension as reports from WHO show that the incidence of hypertension exceeds more than 10% of the worldwide population. In Pakistan, incidence of hypertension is one in every four middle-aged adults (Alamgeer et al. 2013). Several synthetic drugs have been developed for the treatment of hypertension, most of them have showed efficacy but also produce side effects. Herbal medicines therefore, have regain importance because of their ease of availability, less side effects and cost effectiveness (Kalia 2005). In addition, plants consumed in diet, traditionally used medicinal plants have been found useful in management of cardiovascular disease (Hertog et al. 1993) and lowering of blood pressure (Faraji & Tarkhani 1999). Therefore, investigation of plants used in traditional medicine for the treatment of hypertension may serve as one of the most important directions in future for the development of new drugs.

*Ficus carica* Linn. (Moraceae) has been commonly known as ‘Fig’ and is probably a native of southwest Asia that rapidly spread to the Mediterranean region. *Ficus caria* is one of the five plants mentioned in the Quran (Kozubek et al. 2001). It has been widely used for the treatment of cardiovascular diseases in traditional medicine. Despite its traditional usage, there is a lack of data to support the antihypertensive properties of *F. carica*. The present work has investigated the hypotensive effect of aqueous methanol extract of *F. carica* fruit in laboratory animals.

## Material and methods

### Cheimcals used

Adrenaline, calcium chloride and heparin were purchased from Gerhard Buchmann Gmbh^®^, Sigma Chemical Co. (St. Louis, MO) and Leo Pharmaceuticals^®^, respectively. All other chemicals used in this study were of analytical grade and provided by Sigma Chemical Co. (St. Louis, MO).

### Plant material used and preparation of extract

Dried ripe fruits of *F. carica* were purchased during the month of July 2013 from a herbal Store located in Faisalabad. It was identified and authenticated by Dr Ameen Ullah Shah, Taxonomist Department of Biological Sciences, University of Sargodha, Sargodha. A voucher (FC-17-13) has been deposited in the herbarium, Faculty of Pharmacy, University of Sargodha, Sargodha for future reference. The aqueous methanol (70:30) extract of fruit of *F. carica* fruit was prepared by the cold maceration process. Then, it was filtered through Whatman qualitative grade 1 filter paper. The extract was concentrated in a rotary evaporator, under reduced pressure (Titrikou et al. 2007).

### Animals used

Sprague-Dawley rats weighing 220–280 g, albino mice weighing 20–30 g, and healthy adult rabbits, weighing; 1.0–1.5 kg, of either sex were used. The animals were housed in stainless cages under standard laboratory conditions (light period: 8:00 am to 8:00 pm, 21 ± 2 °C, relative humidity 55%). Animals were provided with balanced diet and water *ad libitum*. The study protocol was approved by the Institutional Animal Ethics Committee, Faculty of Pharmacy, University of Sargodha. Experiments comply with the declarations of National Research Council (Clark et al. 1996).

### *In vivo* experiments

#### Screening of different doses and hypotensive effect in normotensive rats

Rats were divided into three groups (*n* = 3). Group I, II, III received 250, 500 and 1000 mg/kg of extracts, respectively. The blood pressure of normotensive rats was noted at 0, 1, 3 and 6 h using non-invasive blood pressure technique (NIBP). The interpretation of SBP and mean blood pressure (MBP) was made from pulse tracings, the diastolic blood pressure (DBP) was calculated from SBP and MBP using equation: DBP = (3MBP–SBP)/2 (Saleem et al. 2005; Yohannes et al. 2010).

#### Evaluation of hypotensive effect in normotensive rats for one month

Rats were randomly divided into two groups (*n* = 3). The group I was served as control and received normal saline while group II served as treated group and treated with 1000 mg/kg for one month. Blood pressure and heart rate of each of these groups were measured at 0, 7, 14, 21 and 28th day using NIBP technique (Saleem et al. 2005).

#### Antihypertensive effect in glucose induced hypertensive rats

Rats were randomly divided into two groups (*n* = 3). Group I was served as control and received 10% glucose solution instead of tap water for consecutive 21 days. In group II, the animals were given 10% glucose solution and 1000 mg/kg extract for consecutive 21 days. Animals were fed on standard diet. Blood pressure and heart rate of each of these groups were measured at 0, 3, 6, 9, 12, 15, 18 and 21 days using NIBP (Saleem et al. 2005).

### *In vitro* experiments

#### Effect on various cardiac parameters using isolated perfused rabbit heart

Experiment was carried out under control flow model. Half an hour before dissection heparin 1000 units were injected intra-peritoneally to rabbits (*n* = 3), to prevent coagulation of blood. The rabbit was killed, and thorax was cut and opened to expose the chest cavity. To prevent ischaemia within 1–2 min the heart was removed from pericardial sac, having 1 cm aorta. Then, it was transferred to an ice-cold oxygenated Krebs–Henseleit solution. After removing excessive tissues the heart was mounted on a Langendroff’s apparatus. Then, aorta was clamped to the cannula with a small blunt artery clip, a ligature was tied rapidly around aorta locking it into groove then artery clip was removed. For the first few minutes the perfusion fluid was passed at an increased flow rate. Later on, the fluid flow rate was decreased and maintained at a suitable flow rate throughout the experiment. Afterward an open tip pressure transducer was attached to the apex of the heart in left ventricle to measure the force of contraction (*g*). The transducers were attached to the power lab and the recordings were measured using computer running chart 5.0 software. After stabilization, the heart was assessed for various cardiac parameters. The preparation was then allowed to equilibrate for 30 min before starting the experiment. The reading was taken as controlled when only the Krebs–Henseleit solution was running. Then, various doses (1 pg, 10 pg, 100 pg, 1 ng, 10 ng, 100 ng, 1 μg, 10 μg, 100 μg, 1 mg, 10 mg) of extract were applied to assess the heart rate and force of contraction. Similarly, different doses of adrenaline and CaCl_2_ were also appraised in order to determine the mechanism of action of crude extract. Each heart served as its own control. Changes in heart rate and force of contraction were expressed as means ± SEM. (Alamgeer et al. 2014).

### Phytochemical investigations

#### Preliminary qualitative phytochemical tests

The extract was screened for various phytochemical constituents (sapoinns, flavonoids, glycosides, tannins, phenolic compounds, steriods, anthraquinones and alkaloids) using standard procedures (Edeoga et al. 2005).

#### Antioxidant evaluation using DPPH free radical scavenging activity

The free radical scavenging capacity was determined by using stable free radical, 2,2-diphenyl-1-picrylhydrazyl (DPPH) according to the method proposed by Brand et al. (1995). The extract was mixed in methanol. Different concentrations of extract (0.5 mL) was added to 3.5 mL of DPPH solution (25 μg/mL). After 30 min the absorbance was measured at 517 nm by spectrophotometer. Ascorbic acid was taken as reference. Percentage inhibition of DPPH free radical scavenging capacity (%I) was calculated on the basis of control reading by following equation:

$$\%I=\left(\left(A_{\mathrm{blank}}-A_{\mathrm{extract}}\right)/A_{\mathrm{blank}}\right)\times 100$$

where, *A*_blank_ is the absorbance of the DPPH solution with methanol and *A*_extract_ is the absorbance of solution with extract.

#### Determination of total flavonoid content (TF)

The extract (0.5 mL) was mixed with distilled water (2.2 mL) and 5% of NaNO_2_ solution (0.15 mL). After 6 min, 10% AlCl_3_·6H_2_O solution (0.3 mL) was added and allowed to stand for 5 min. Then, 1 M NaOH (1 mL) was added. After vortexing the mixture, absorbance was measured at 510 nm using spectrophotometer. Flavonols in extract were expressed as quercetin equivalents. Quercetin (Sigma, Germany) was used to perform the calibration curve (standard solutions of 6.25, 12.5, 25.0, 50.0, 80.0 and 100.0 μg/mL in 80% ethanol (V/V) (Chang et al. 2002).

#### Determination of total phenolics contents (TPC)

The amount of TPC was evaluated by Folin–Ciocalteu reagent (Chaovanalikit & Wrolstad 2008). Extract (50 mg) was mixed with Folin–Ciocalteu reagent (0.5 mL) and deionized water (7.5 mL). After 10 min, 20% sodium carbonate (w/v) (1.5 mL) was added. Mixture was heated for 20 min in water bath at 40 °C and then cooled. Absorbance was measured at 755 nm using spectrophotometer. The amount of TPC was calculated by using a gallic acid calibration curve within the range of 10–100 ppm (*R* ^2^ = 0.9986). The result were expressed as gallic acid equivalents (GAE) g/100 g of dry matter. All samples were analyzed thrice.

#### Determination of phenolic profile with HPLC-DAD

An HPLC analysis was performed using (model LC-10A, Shimadzu, Japan), equipped with two LC-10 AT pumps, SCL-10 A system control unit, Rheodyne injector, CTO-10A column oven, SPD-10A UV-Vis detector and data acquisition class LC-10 software were used. A 20 μL volume of the filtered sample was injected into an analytical Supelco (Supelco Inc., Supelco Park, Bellefonte, PA) ODS reverse phase (C18) column (250 × 4.6 mm; 5 μm particle size). Two solvent systems A: contained 3% triflouroacetic acid and B: contained acetonoitrile and methanol (80:20 v/v) were used. The chromatographic separation was performed by isocratic elution of the mobile phase (mixture of solvent A and B (50:50 v/v), which was filtered under vacuum through a 0.45 μm membrane before use) at a flow rate of 1.0 mL per min at 30 °C. Detection was performed at a wavelength of 280 nm (Sultana et al. 2008).

### Statistical analysis

The results are expressed as means ± standard error of mean (SEM). Results were statistically analyzed using one-way ANOVA and two-way ANOVA as required followed by Dunnet’s post. The data with *p* values 95% (*p* < 0.05) was considered statistically significant. Graph pad prism version 5.00 was the software used for statistical analysis.

## Results

### Screening of different doses and hypotensive effect in normotensive rats

**Figure 1.**
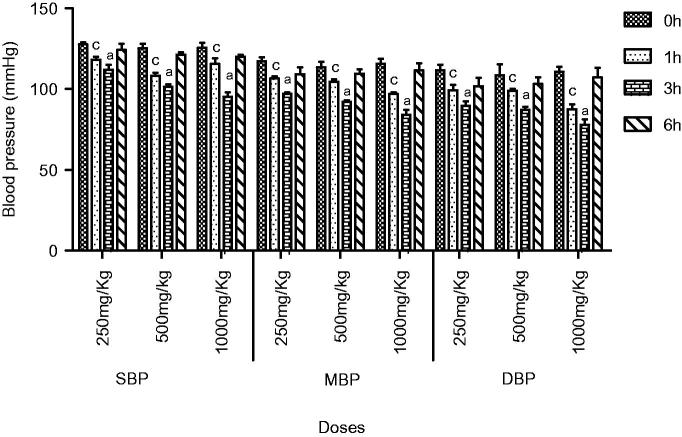
Effect of screening of various doses of extract on systolic blood pressure (mmHg), mean blood pressure and diastolic blood pressure in normotensive rats. The results are stated as Mean ± SEM, where c = (*p* < 0.05), a = (*p* < 0.001) vs. control (0 h).

**Figure 2.**
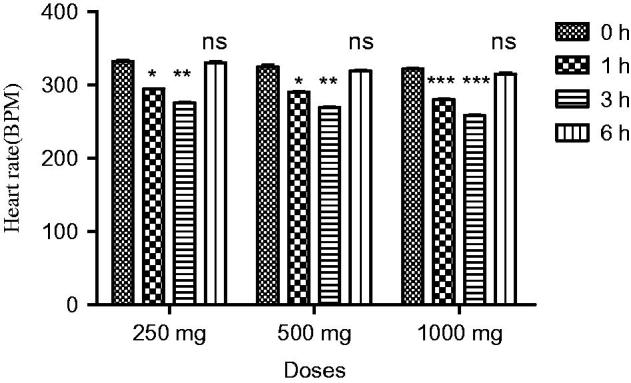
Effect of screening of various doses of extract on heart rate of normotensive rats, where, *** = (*p* < 0.001), and ns = non-significant vs. control (0 h).

In normotensive rats, extract at all doses showed a significant decrease in SBP (*p* < 0.05) and MBP (*p* < 0.05) at the first hour. Similarly, a highly significant reduction in SBP (*p* < 0.001) and MBP was observed at third hour. A significant decrease in DBP was observed (*p* < 0.05) at the first and (*p* < 0.001) at third hour. The hypotensive effect of extract started decreasing after 3 h. The extract demonstrated a highly significant decrease in heart rate (*p* < 0.01) at the first and third hour, while a slight reduction in blood pressure was observed at the sixth hour. A maximum decrease in all parameters was observed at 1000 mg/kg (Figures 1 and 2).

### Evaluation of hypotensive effect in normotensive rats for one month

**Figure 3.**
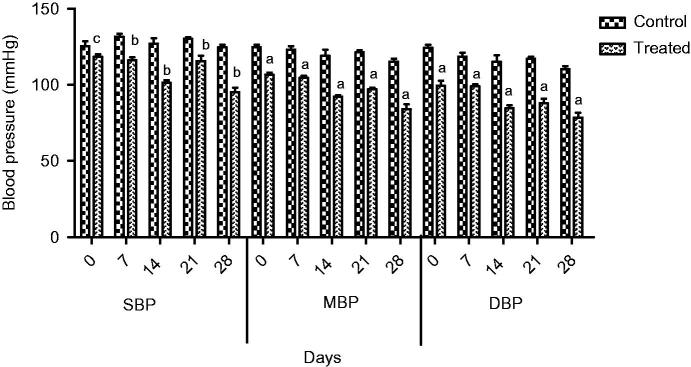
Effect of extract on SBP,MBP and DBP of normotensive rats. Results are presented as Mean ± SEM where c = (*p* < 0.05), b = (*p* < 0.01) and a = (*p* < 0.001) vs. control.

**Figure 4.**
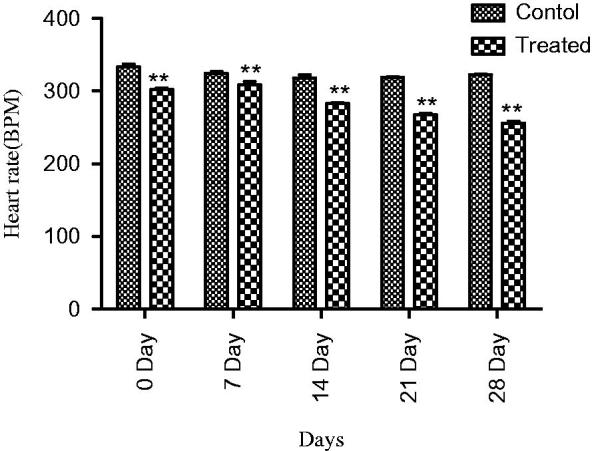
Effect of extract on heart rate of normotensive rats, where ** = (*p* < 0.01) vs. control.

In normotensive rats, extract at 1000 mg/kg dose showed a significant decrease (*p* < 0.01) in SBP, and MBP and heart rate at 7, 14, 21 and 28 days, a maximum was observed at the fourth week. Similarly, a significant fall in DBP (*p* < 0.001) was observed at 7, 14, 21 and 28 days (Figures 3 and 4).

### Evaluation of antihypertensive effect in glucose-induced hypertensive rats

**Figure 5.**
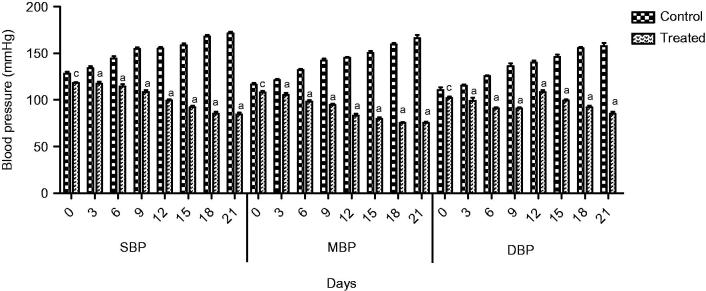
Effect of extract on SBP, MBP and DBP of glucose treated hypertensive rats. Where c = (*p* < 0.05) and a = (*p* < 0.001) vs. control.

**Figure 6.**
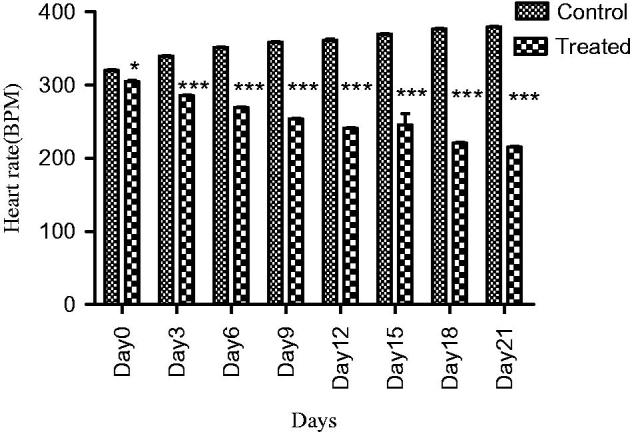
Effect of extract on heart rate of glucose treated hypertensive rats, where * = (*p* < 0.05), and *** = (*p* < 0.001) vs. control.

The extract at a dose of 1000 mg/kg significantly (*p* < 0.001) prevented the rise in SBP, MBP, DBP and heart rate of the glucose-treated hypertensive rats with more pronounced effects at week 3 compared to control (Figures 5 and 6).

### Effect on various cardiac parameters using isolated perfused rabbit heart

In isolated heart preparation, the extract produced a dose-dependent and significant decrease in heart rate and force of contraction in all the given doses (0.000000001–0.01 mg/mL) but at the dose of 0.1–10 mg/mL the change in force of contraction become non-significant. While the dose of 0.001 mg/mL was selected for determination of possible mechanism of action (Table 1).

**Table 1.** Effect of aqueous-methanolic extract of *Ficus carica* fruit on various cardiac parameters of isolated rabbit heart.

	Force of contraction (g)		Heart rate (BPM)	
Dose	Mean ± SEM	% Change from control	Mean ± SEM	% Change from control
Control	2.83 ± 0.08	0.00	126.0 ± 1.45	0.00
1 pg/mL	3.00 ± 0.11^ns^	6.0	125.0 ± 0.88^ns^	0.2
10 pg/mL	2.97 ± 0.12^ns^	4.9	133.0 ± 1.53*	5.8
100 pg/mL	2.70 ± 0.11^ns^	4.5	111.0 ± 1.15***	11.6
1 ng/mL	2.63 ± 0.08^ns^	7.0	96.7 ± 1.45***	23.0
10 ng/mL	2.23 ± 0.08**	21.2	88.6 ± 1.20***	29.3
100 ng/mL	1.93 ± 0.12**	31.8	79.3 ± 1.45***	36.7
1 μg/mL	1.67 ± 0.08***	40.9	71.0 ± 1.53***	43.4
10 μg/mL	2.03 ± 0.14**	28.2	84.0 ± 1.73***	33.0
100 μg/mL	2.63 ± 0.08^ns^	7.06	90.7 ± 1.20***	27.7
1 mg/mL	3.07 ± 0.14^ns^	8.4	101.0 ± 1.15***	19.6
10 mg/mL	3.17 ± 0.20^ns^	11.7	110.0 ± 1.20***	12.1

Values are expressed as means ± SEM (*n* = 3).

* *p* < 0.05).

** *p* < 0.01).

*** *p* < 0.001. ns: non-significant vs. control.

### Effect of adrenaline (10^−5^ M) in the presence of aqueous-methanol extract

The results revealed that extract (0.001 mg/mL) did not significantly blocked the effects of adrenaline (10^−5^ M) on isolated rabbit hearts. Adrenaline (10^−5^ M) produced significant change in force of contraction and heart rate in the presence of extract.

### Effect of calcium chloride (10^−5^ M) in the presence of aqueous-methanol extract

To find out the effect of extract on calcium channels, the effects of CaCl_2_ on various cardiac parameters were studied both in the presence and absence of extract (0.001 mg/mL). Calcium chloride (10^−5^ M) produced non-significant change in force of contraction and heart rate in the presence of extract thus the results are not shown.

### Phytochemical investigations

#### Organoleptic and macro-morphological evaluation

The fruit of *F. carica* is technically a synconium (a fleshy hollow receptacle having a small opening at the apex partly closed by small scales). It may be obovoid, turbinate, or pear-shaped, 1–4 inches long. The matured ‘fruit’ has a tough peel (pure green, green suffused with brown, brown or purple), often cracking upon ripeness, and exposing the pulp beneath having pleasant odour and typically taste sweet. The interior is a white inner rind containing a seed mass bound with jelly-like flesh.

#### Preliminary qualitative phytochemical tests

The phytochemical screening of extract confirmed the presence of different secondary metabolites such as saponins, flavonoids, glycosides, tannins, phenolic compounds, steroids, anthraquinones and alkaloids by using standard procedures.

#### Antioxidant evaluation using DPPH free radical scavenging activity

**Figure 7.**
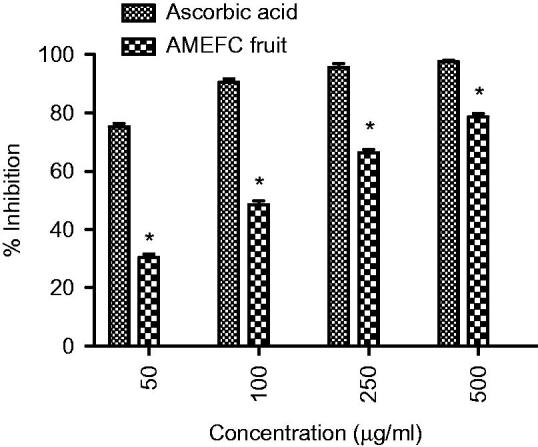
Effect of extract on DPPH scavenging activity. Where * = (*p* < 0.05) vs. ascorbic acid.

Increase of 1% in concentration-dependent manner was observed in the extract (Figure 7).

#### Determination of total flavonoids (TF) and total phenolic content (TPC)

Results of AlCl_3_ assay of aqueous methanol extract obtained from fruits of *F. carica* indicate the presence of total flavonoids that is 538.20 ± 1.17% W/W. Additionally, total phenolic contents were assessed by Folin–Ciocalteu reagent assay in value of 31.88 ± 1.48% W/W.

#### Determination of phenolic profile with HPLC-DAD

**Figure 8.**
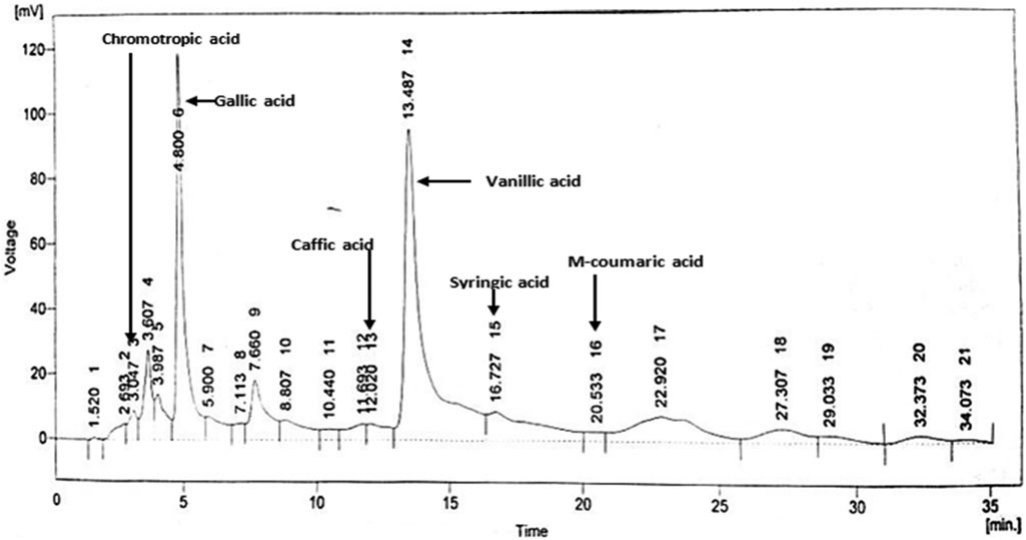
Chromatogram presenting compounds in AMEFC fruit at 280 nm.

Analysis of phenolic compounds by HPLC-DAD system of extract revealed the presence of chromotropic acid, quercetin, gallic acid, caffic acid, vanillic acid, syringic acid, *m*-coumaric acid in chromatogram (Figure 8).

## Discussion

Traditional drugs have been used from primordial times. In this milieu, aboriginal drugs considered to be of great importance from both economic and professional point of view (Tapsell et al. 2006). The searching for food that decreases the risk for cardiovascular diseases brought nuts and dry fruits into lamb light for experimentation. This identified its usefulness for protection against heart diseases. *Ficus carica* (Fig), one of the commonest dry fruit, has been used from centuries (Sabate 1999) both for nutritional as well as medicinal purposes. However, the efficacy of aqueous methanol extract of *F.carica* fruit as an antihypertensive agent has never been investigated. On this basis the current investigation was conducted to evaluate the cardiovascular and antihypertensive effects of *F. carica* fruit. The main finding of this study was that the aqueous methanol extract obtained from fruit of *F. carica* was able to decrease blood pressure both in normotensive as well as glucose-treated hypertensive rats. It has been reported that abnormal glucose level commonly accompanies with hypertension and this alliance is known as insulin-resistance syndrome (Ferrannini et al. 1987). In view of that, many experimental studies presented that fructose and glucose contributes to the increase in blood pressure (Ranganath et al. 1998; Midaoui & Champlain 2002) by numerous mechanisms including fluid volume expansion and sodium retention (Baum 1987), dyslipidaemia (Reaven & Ho 1991), endothelial dysfunction (Tomiyama et al. 2000), increase in oxidative stress (Cai & Harisson 2000) and stimulation of sympathetic system (Bunag et al. 1983). In present study, we used glucose-induced hypertension model to investigate antihypertensive effect of aqueous methanol extract of *F. carica,* for this purpose 10% glucose solution was used in drinking water which induced a significant increase in blood pressure of rats. Pharmacological investigation of aqueous methanol extract of *F. carica* fruit showed a dose-dependent decrease in the SBP, MBP, DBP and heart rate in both the normotensive and glucose-treated hypertensive albino rats. The results of screening of different doses such as 250, 500 and 1000 mg/kg showed that decreases in all the above parameters were more pronounced at 1000 mg/kg (Figures 1–3). The hypotensive effect of extract was more pronounced in glucose-induced hypertensive rats as compared to normotensive rats. This is in line with previous findings that hypertensive rats appear to have more pronounced response to hypotensive agents than normotensive rats (Bunang et al. 1975). It has been reported that plants rich in polyphenols having an antioxidant effect, which improves endothelial dysfunction through increase NO formation, decrease LDL formation, increase prostacyclin formation, increase endothelium-derived hyperpolarizing factor (EDHF)-mediated vasorelaxation and decrease Endothelin-1 production. So, the polyphenols concerning vasorelaxation beneficial effects have been attributed to blood pressure reducing properties in rodents (Duarte et al. 2001, 2002; Jalili et al. 2006). In the current study, the crude extract showed the presence of flavonoids, and good antioxidant potential (Figure 7). Thus, the antihypertensive effect of *F. carica* L fruit may be due to the antioxidant effect of polyphenols.

Further to scrutinize the possible reason for antihypertensive effect, *in vitro* study was conducted on isolated perfused rabbit heart using Langendroff’s technique. The crude extract produced a transient but significant decrease in force of contraction and heart rate (Table 1). The dose of 0.001 mg/mL of crude extract, which have produced the maximum effect (*p* < 0.001) was selected for elucidation of mechanism of action. For determining the beta-adrenergic blocking activity adrenaline was used. The results of experiment showed that crude extract did not block the stimulatory effect of adrenaline (10^−5^ M) showing no involvement of beta-adrenergic receptor for its action. Similarly, the calcium channel blocking activity of crude extract was checked by calcium chloride (10^−5^ M), results showed that the stimulatory effect of calcium chloride was not affected by its presence. So, it is inferred from these experiments that crude extract produced its cardioinhibitory effect by some other mechanisms.

Considering the present data on *F. carica* fruit, the amount of total flavonoid content was determined spectrophotometerically. Results of several studies showed good interrelationship between the total phenolic content and antioxidant activity of plant extract (Moure et al. 2001). The total phenolic content was determined with standard gallic acid and results represented in terms of gallic acid equivalents (mg GAE/g DW). In order to determine the phenolic compounds, HPLC-DAD technique was used. The results showed the presence of quercetin, gallic acid, caffeic acid, vanillic acid, syringic acid, *m*-coumaric acid as shown in chromatogram (Figure 8).

## Conclusions

It is concluded from the present study that cardioinhibitory, antihypertensive and diuretic effect of *F. carica* fruit may be due to the presence of flavonoids, phenols and potassium through different mechanisms. The results of experimental data propose that *F. carica* fruit may reduce coronary heart disease risk in hypertensive subjects, ameliorate endurance in patients with heart failure, and may influence coronary ischaemia and reperfusion injury. However, further studies are required to isolate these compounds and elucidate their exact mechanism of action.
